# Development and validation of a predictive risk factor model for epidural re-siting in women undergoing labour epidural analgesia: a retrospective cohort study

**DOI:** 10.1186/s12871-018-0638-x

**Published:** 2018-11-29

**Authors:** John Song En Lee, Rehena Sultana, Nian Lin Reena Han, Alex Tiong Heng Sia, Ban Leong Sng

**Affiliations:** 1 Department of Women’s Anaesthesia, KK Women’s and Children’s Hospital, 100 Bukit Timah Road, Singapore, 229899 Singapore; 2 Centre for Quantitative Medicine, Duke-NUS Medical School, 8 College Road, Singapore, 169857 Singapore; 3 Division of Clinical Support Services, KK Women’s and Children’s Hospital, 100 Bukit Timah Road, Singapore, 229899 Singapore; 4 Anaesthesiology and Perioperative Sciences Academic Clinical Program, Duke-NUS Medical School, 8 College Road, Singapore, Singapore

**Keywords:** Epidural, Labour, Re-siting, Predictive model

## Abstract

**Background:**

Epidural catheter re-siting in parturients receiving labour epidural analgesia is distressing to the parturient and places them at increased complications from a repeat procedure. The aim of this study was to develop and validate a clinical risk factor model to predict the incidence of epidural catheter re-siting in labour analgesia.

**Methods:**

The data from parturients that received labour epidural analgesia in our centre during 2014–2015 was used to develop a predictive model for epidural catheter re-siting during labour analgesia. Multivariate logistic regression analysis was used to identify factors that were predictive of epidural catheter re-siting. The forward, backward and stepwise variable selection methods were applied to build a predictive model, which was internally validated. The final multivariate model was externally validated with the data collected from 10,170 parturients during 2012–2013 in our centre.

**Results:**

Ninety-three (0.88%) parturients in 2014–2015 required re-siting of their epidural catheter. The training data set included 7439 paturients in 2014–2015. A higher incidence of breakthrough pain (OR = 4.42), increasing age (OR = 1.07), an increased pain score post-epidural catheter insertion (OR = 1.35) and problems such as inability to obtain cerebrospinal fluid in combined spinal epidural technique (OR = 2.06) and venous puncture (OR = 1.70) were found to be significantly predictive of epidural catheter re-siting, while spontaneous onset of labour (OR = 0.31) was found to be protective. The predictive model was validated internally on a further 3189 paturients from the data of 2014–2015 and externally on 10,170 paturients from the data of 2012–2013. Predictive accuracy of the model based on C-statistic were 0.89 (0.86, 0.93) and 0.92 (0.88, 0.97) for training and internal validation data respectively. Similarly, predictive accuracy in terms of C-statistic was 0.89 (0.86, 0.92) based on 2012–2013 data.

**Conclusion:**

Our predictive model of epidural re-siting in parturients receiving labour epidural analgesia could provide timely identification of high-risk paturients required epidural re-siting.

## Background

Inadequate relief of labour pain can be distressing to the parturient. Current pain relief modalities could include epidural analgesia, nitrous oxide (Entonox), intramuscular pethidine and intravenous remifentanil [1, 2]. Epidural analgesia is the gold standard for labour pain relief as it provides superior pain relief with minimal neonatal adverse effects [3]. However, a poorly functioning epidural catheter that does not provide adequate pain relief may require a repeat invasive procedure for re-siting. This would lead to further distress to the parturient and may place them at additional risk for complications from another epidural catheter placement [4].

Patient, obstetric and anaesthetic factors have been associated with inadequate pain relief with labour epidural analgesia [5–8]. In a retrospective cohort study conducted in our centre from 2012 to 2013, the incidence of epidural catheter re-siting in parturients with epidural labour analgesia was found to be 0.85% [4]. We identified independent association factors that were associated with epidural catheter re-siting, which included a greater quantity of dinoprostone (Prostin E2) used for induction of labour, a longer time taken to perform the neuraxial blockade, a higher incidence of breakthrough pain, requirement for Caesarean section for delivery and complications associated with epidural analgesia such as venous puncture as well as hypotension and shivering [4]. The area under curve (AUC) of the receiver operating characteristic (ROC) curve for this multivariate model was 0.894.

Although active management such as epidural supplementation could increase the success rate of epidural analgesia, the timely recognition of the modifiable predictive risk factors of epidural catheter re-siting could allow targeted management, thereby potentially reducing the incidence of epidural catheter re-siting. Thus, the objective of this study was to identify predictive factors for epidural catheter re-siting, evaluate the predictive accuracy of these set of predictors (including demographic and clinical variables), as well as to validate them internally and externally for predicting the risk of epidural catheter re-siting. The latest 2014–2015 data was used to develop a predictive model, which was subsequently externally validated with the pre-existing 2012–2013 data from our previous study.

## Methods

This is a retrospective cohort study that involved the collection of data of all parturients that received neuraxial analgesia (combined spinal epidural (CSE) or epidural analgesia) in KK Women’s and Children’s Hospital (KKH), Singapore, between January 2012 and December 2015. We included all women who underwent labour epidural analgesia at KKH and there was no specific sampling, inclusion and exclusion criteria as the database included all women. This study received approval by the Singhealth Centralized Institutional Review Board (CIRB) (Ref: 2017/2023 (2014–2015 data) and 2012/259/D (2012–2013 data)).

Our centre maintains an electronic database of details of labour neuraxial analgesia received by parturients, which is collated from the labour neuraxial analgesia forms. The electronic records of 10,628 parturients that received labour neuraxial analgesia from January 2014 to December 2015 and 10,170 paturients from January 2012 to December 2013 were obtained from the electronic database. Missing and outlier values were identified and the corresponding forms had values cross-checked and amended as appropriately.

### Labour neuraxial analgesia regime

The type of labour neuraxial analgesia performed (CSE or plain epidural analgesia) and the local anaesthetic regimens used for labour analgesia was at the discretion of the attending anaesthesiologist on duty. For the induction of labour analgesia, a typical regimen of 2 mg ropivacaine and 15 mcg fentanyl was commonly administered intrathecally for a CSE and 10–20 ml of ropivcaine 0.2% for plain epidural analgesia. For the maintenance of labour analgesia, a basal infusion rate of 5 to 12 ml/hr. of local anaesthetic 0.1 to 0.125% ropivacaine or bupivacaine with fentanyl 2mcg/ml was commonly used, with a bolus of 5 ml for each successful patient initiated demand.

### Data collection

Maternal demographic data, including age, race, weight (kg) and height (cm) was recorded. Obstetric data collected included parity, use of dinoprostone (Prostin E2) for induction of labour, cervical dilatation and use of oxytocin for labour augmentation pre-neuraxial blockade, duration of second stage of labour as well as mode of delivery.

Anaesthetic data included the American Society of Anaesthesiologists physical classification status, use of intramuscular pethidine or nitrous oxide (Entonox) for analgesia pre-neuraxial blockade as well as pre- and post-procedure pain scores (measured on a Visual Analogue Scale of 0–10) [9]. The seniority of the attending anaesthesiologist (Specialist or Resident/Medical Officer), type of analgesia (CSE or plain epidural analgesia), type and concentration of local anaesthetic used for induction and maintenance of labour analgesia, level of insertion, number of attempts, time taken, number of anaesthesiologists that attempted neuraxial blockade before successful placement and total volume of local anaesthetic infused at delivery were recorded.

Side effects and complications as documented by either nursing staff or anaesthesiologist such as hypotension (> 20% drop in systolic blood pressure) and fetal bradycardia post-neuraxial blockade, inability to obtain cerebrospinal fluid (CSF) from the spinal needle inserted through the epidural needle in the CSE technique, venous puncture, inability to pass the epidural catheter through the epidural needle, paresthesia, dural puncture, symptoms of local anaesthetic toxicity, high blockade (sensory level of T1 and above), dislodgement of the epidural catheter, shivering, pruritus, nausea, vomiting and presence of breakthrough pain were also obtained from the electronic database.

Breakthrough pain was defined as a maternal complaint of pain that required the attending anaesthesiologist to administer an additional bolus of local anaesthetic agent. A common regimen would be 0.2–0.3% ropivacaine 5-10 ml, with or without epidural opioids (fentanyl 50 mcg). The reason for inadequate pain relief (inadequate level, unilateral blockade, patchy block, perineal pain or back pain), cervical dilatation, oxytocin infusion rate (ml/hr), the volume and concentration of local anaesthetic agent administered and the pain scores before and after the additional administration of an epidural bolus were documented. Epidural catheter re-siting was defined as the need to remove the epidural catheter and perform another neuraxial block procedure. The decision to re-site the epidural catheter was at the discretion of the attending anaesthesiologist on duty and was recorded.

### Study outcome

The study outcome was the incidence of epidural catheter re-siting in all parturients that received labour neuraxial analgesia (CSE or plain epidural analgesia)—this was treated as binary data with categories as “*requiring epidural catheter re-siting*” or “*not requiring epidural catheter re-siting*”.

### Statistical analysis

Logistic regression model was used to find the effect of each variable on epidural catheter re-siting. Variables available prior to the epidural catheter re-siting procedure assumed to be associated were maternal age (years), race, maternal weight (kg), maternal height (cm), maternal BMI (kg/m^2^), quantity of dinoprostone (prostin E2), cervical dilatation pre-neuraxial blockade (cm), mode of delivery, spontaneous labour onset, labour onset: artificial rupture of membranes, labour onset: dinoprostone insertion, type of anaesthetic technique, number of anaesthetists, time taken for neuraxial block (min), post epidural pain score, total volume of local anaesthetic infused at delivery (ml), incidence of breakthrough pain, hypotension, shivering, inability to obtain CSF in the CSE technique, venous puncture and inability to pass catheter through epidural needle. Variables with *p*–value < 0.3 on the univariate logistic regression analysis or that were clinically meaningful were included in the multivariable logistic regression model. Several clinical interactions were also considered. The union of the variables from forward, backward and stepwise variables selection methods were used to finalize the list of variables in the multivariable model with entry and stay criteria as 0.9 and 0.2 respectively. Improvement in model performance through the addition of new candidate variables in multivariable logistic regression models was tested using concordance statistics (C-Statistics) with Akaike information criterion (AIC). AIC measures both the accuracy and complexity of a model. For a given situation, a model with lower AIC generally has the better generalizability [10]. The logistic regression model develops a score which is a linear combination of selected variables. This score can be converted to the estimated probability of epidural catheter re-siting using the following formula:

$$ \mathrm{Estimated}\ \mathrm{probability}=\frac{{\mathrm{e}}^{\mathrm{Score}}}{1+{\mathrm{e}}^{\mathrm{Score}}},\mathrm{where}\ \mathrm{e}\ \mathrm{is}\ \mathrm{natural}\ \mathrm{exponential}. $$

Sensitivity, specificity, positive predictive value and negative predictive value were also calculated. To obtain realistic and generalizable accuracy estimates, we first randomly split data from the 2014–2015 set with parturients that received labour neuraxial analgesia in our centre into two sets: 70 and 30% parturients was referred as training data and internal validation data, respectively. The training data was used to finalize the predictive model. The predictive accuracy of the model based on the internal validation data was assessed using C – statistic. This model’s predictive accuracy and robustness was further assessed again using the 2012–2013 data set on parturients that received labour neuraxial analgesia in our centre. Point and interval estimates of the C-Statistics were generated.

Parturients’ demographic, obstetric and anaesthetic data were also summarized based on their status of epidural catheter re-siting: “*requiring epidural catheter re-siting*” or “*not requiring epidural catheter re-siting*”. Continuous and categorical variables were summarized as frequency (percentage) or mean (standard deviation (SD)) respectively. Data was summarized for all available data sets: training, internal validation and external validation data. Association from logistic regression model was expressed as β coefficient along with associated 95% confidence interval (95%CI). We have also expressed this association in terms of odds ratio (OR). A *p* – value < 0.05 was considered as statistical significance for a two-sided test. Analysis was done using SAS version 9.3 software (SAS Institute Inc.; Cary, NC, USA).

## Results

**Fig. 1:**
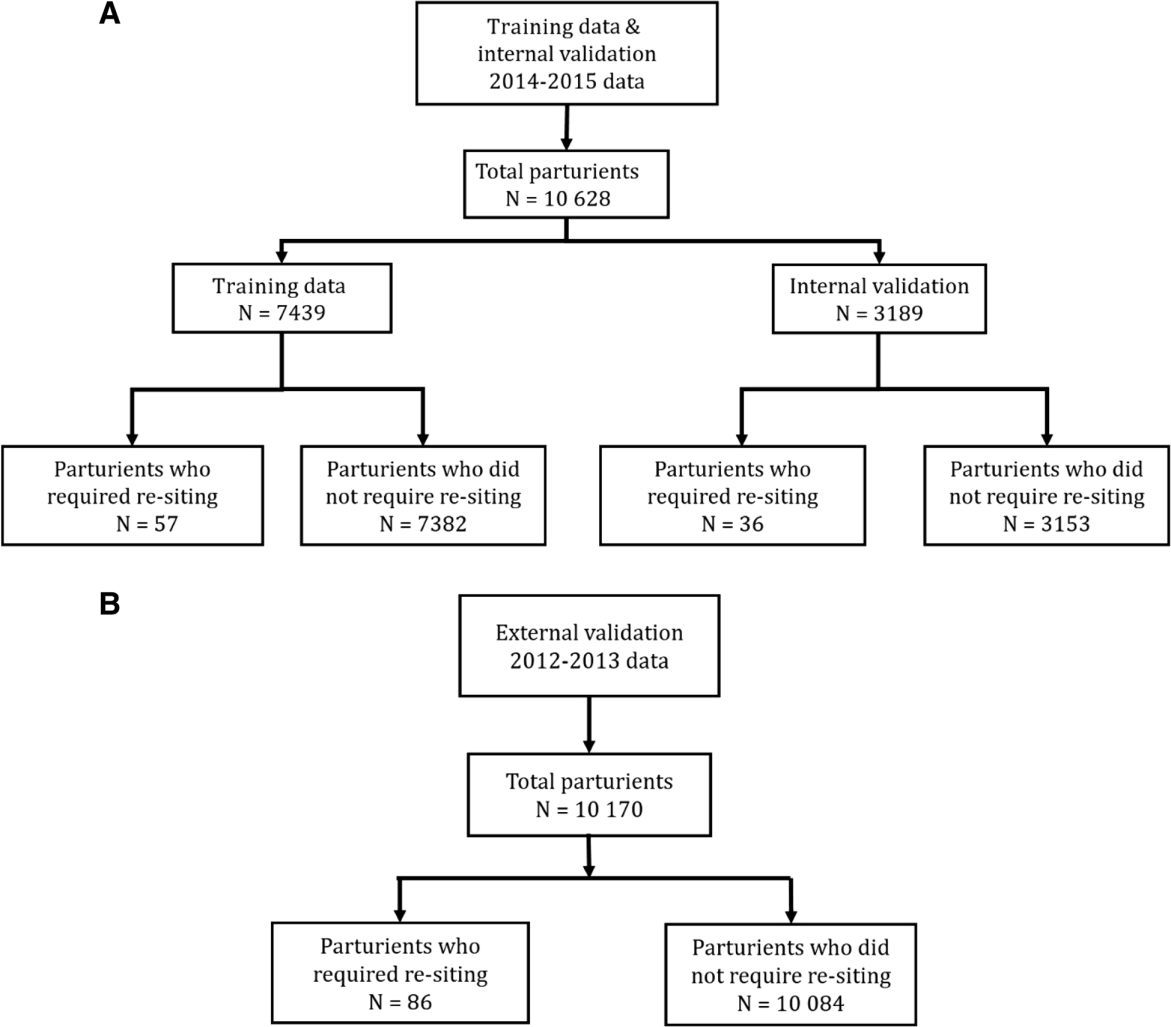
Study flow chart. **a** The 2014–2015 data set for training and internal validation. **b** The 2012–2013 data set for external validation

From 1 January 2014 to 31 December 2015, 10,628 parturients received labour epidural analgesia at our centre. Ninety-three (0.88%) parturients required re-siting of their epidural catheters. From January 2012 to December 2013, there were 10,170 parturients that received labour epidural analgesia and 86 (0.85%) paturients required epidural catheter re-siting. (Fig. 1).

### Training and internal validation data

The 2014–2015 data were randomly divided in training and internal validation data. Training data comprised of 7439 (70%) parturients was used to develop predictive model while internal validation data comprised of 3189 (30%) parturients to check the predictive property of the predictive model. There were 57 (0.77%), 36 (1.13%) and 86 (0.85%) cases of epidural catheter re-siting in the training, internal validation and external validation data respectively. 6930 (93.2%), 2958 (92.7%) and 9533 (94.7%) parturients received a CSE in the training, internal validation and external validation data respectively. Characteristics of the three data are shown in Table 1.

**Table 1:** Patient’s demographic, clinical and anesthetic characteristics of patients in training, internal and external validation data

Characteristics	Training data (*N* = 7439)		Internal validation data (*N* = 3189)		External validation data (*N* = 10,170)	
	Epidural re-siting		Epidural re-siting		Epidural re-siting	
	No (*N* = 7382)	Yes (*N* = 57)	No (*N* = 3153)	Yes (*N* = 36)	No (N = 10,084)	Yes (*N* = 86)
Demographic Data						
Age (years), mean (SD)	30.2 (5.0)	31.2 (4.3)	30.2 (4.9)	30.0 (4.6)	29.9 (5.0)	29.1 (5.0)
Race, n(%)						
Chinese	3421 (46.3)	22 (38.6)	1488 (47.2)	11 (30.6)	4805 (47.6)	31 (36.0)
Indian	956 (13.0)	9 (15.8)	407 (12.9)	8 (22.2)	1263 (12.5)	14 (16.3)
Malay	1657 (22.4)	15 (26.3)	648 (20.6)	11 (30.6)	2361 (23.4)	19 (22.1)
Others	1348 (18.3)	11 (19.3)	610 (19.3)	6 (16.7)	1655 (16.4)	22 (25.6)
Maternal weight (kg), mean (SD)	68.2 (13.1)	70.7 (12.1)	68.0 (12.9)	69.2 (12.4)	69.2 (13.0)	71.8 (14.3)
Maternal height (cm), mean (SD)	160 (6)	160 (6)	160 (5)	160 (6)	158.7 (8.5)	157.6 (6.3)
Maternal BMI (kg/m^2^), mean (SD)	27.1 (6.0)	28.1 (4.2)	27.0 (4.8)	28.1 (4.6)	27.5 (5.5)	28.9 (5.1)
Obstetric Data						
Quantity of dinoprostone, mean (SD)	0.4 (0.8)	0.5 (0.8)	0.4 (0.7)	0.6 (1.1)	1.2 (2.7)	2.1 (2.7)
Cervical dilatation pre-neuraxial blockade (cm), mean (SD)	3.5 (1.0)	3.2 (0.8)	3.5 (1.0)	3.0 (0.8)	3.5 (1.2)	3.1 (0.9)
Mode of delivery, n (%)						
Instrumental delivery	738 (10.0)	2 (3.5)	293 (9.3)	8 (22.2)	1017 (10.1)	11 (12.8)
Caesarean Section	1288 (17.5)	22 (38.6)	498 (15.8)	11 (30.6)	1675 (16.7)	29 (33.7)
Normal vaginal delivery	5355 (72.6)	33 (57.9)	2362 (74.9)	17 (47.2)	7345 (73.2)	46 (53.5)
Spontaneous labour onset, n (%)						
No	3532 (47.8)	31 (54.4)	1537 (48.7)	17 (47.2)	5293 (52.5)	46 (53.5)
Yes	3850 (52.2)	26 (45.6)	1616 (51.3)	19 (52.8)	4791 (47.5)	40 (46.5)
Labour onset, artificial rupture of membranes, n(%)						
No	5530 (74.9)	46 (80.7)	2348 (74.5)	31 (86.1)	7091 (70.3)	71 (82.6)
Yes	1852 (25.1)	11 (19.3)	805 (25.5)	5 (13.9)	2993 (29.7)	15 (17.4)
Labour onset, dinoprostone insertion, n (%)						
No	5435 (73.6)	37 (64.9)	2309 (3.2)	23 (63.9)	7133 (70.7)	49 (57.0)
Yes	1947 (26.4)	20 (35.1)	844 (26.8)	13 (36.1)	2951 (29.3)	37 (43.0)
Anaesthetic Data						
Type of anaesthetic technique, n (%)						
CSE	6878 (93.2)	52 (91.2)	2929 (92.9)	29 (80.6)	9548 (94.8)	75 (87.2)
Epidural	504 (6.8)	5 (8.8)	224 (7.1)	7 (19.4)	526 (5.2)	11 (12.8)
Number of anaesthetists, n (%)						
1	7228 (97.9)	56 (98.2)	3106 (98.5)	35 (97.2)	9923 (98.5)	84 (97.7)
2	152 (2.1)	1 (1.8)	46 (1.5)	1 (2.8)	152 (1.5)	1 (1.2)
3	2 (0.0)	0 (0.0)	1 (0.0)	0 (0.0)	3 (0.0)	1 (1.2)
Time taken for neuraxial block (min), mean (SD)	7.9 (5.9)	8.2 (6.0)	7.6 (5.2)	10.4 (7.8)	6.9 (4.8)	9.2 (7.2)
Post epidural pain score, median (IQR)	0.0 (0.0)	0.0 (0.0)	0 (0.0)	0.0 (1.0)	0.0 (0.0)	0.0 (0.0)
Total volume of local anaesthetic infused at delivery (ml), mean (SD)	57.1 (44.1)	92.6 (69.5)	57.1 (44.0)	87.6 (62.0)	56.3 (43.1)	87.3 (59.5)
Incidence of breakthrough pain, n (%)						
Yes	994 (13.5)	43 (75.4)	430 (13.6)	31 (86.1)	1386 (13.7)	68 (79.1)
No	6388 (86.5)	14 (24.6)	2723 (86.4)	5 (13.9)	8698 (86.3)	18 (20.9)
Hypotension, n (%)						
Yes	50 (0.7)	0 (0.0)	16 (0.5)	0 (0.0)	77 (0.8)	4 (4.7)
No	7332 (99.3)	57 (100.0)	3137 (99.5)	36 (100.0)	10,007 (99.2)	82 (95.3)
Shivering, n (%)						
Yes	1681 (22.8)	13 (22.8)	676 (21.4)	8 (22.2)	2526 (25.0)	32 (37.2)
No	5701 (77.2)	44 (77.2)	2477 (78.6)	28 (77.8)	7558 (75)	54 (62.8)
Inability to obtain CSF in the CSE technique, n (%)						
Yes	113 (1.5)	5 (8.8)	48 (1.5)	5 (13.9)	169 (1.7)	7 (8.1)
No	7269 (98.5)	52 (91.2)	3105 (98.5)	31 (86.1)	9915 (98.3)	79 (91.9)
Venous puncture, n (%)						
Yes	219 (3.0)	6 (10.5)	104 (3.3)	8 (22.2)	318 (3.2)	10 (11.6)
No	7163 (97.0)	51 (89.5)	3049 (96.7)	28 (77.8)	9766 (96.8)	76 (88.4)
Inability to pass catheter through epidural needle, n(%)						
Yes	26 (0.4)	1 (1.8)	9 (0.3)	0 (0.0)	5 (0.0)	2 (2.3)
No	7356 (99.6)	56 (98.2)	3144 (99.7)	36 (100.0)	10,079 (100.0)	84 (97.7)

*BMI* Body mass index, *CSE* Combined spinal epidural

**Fig. 2:**
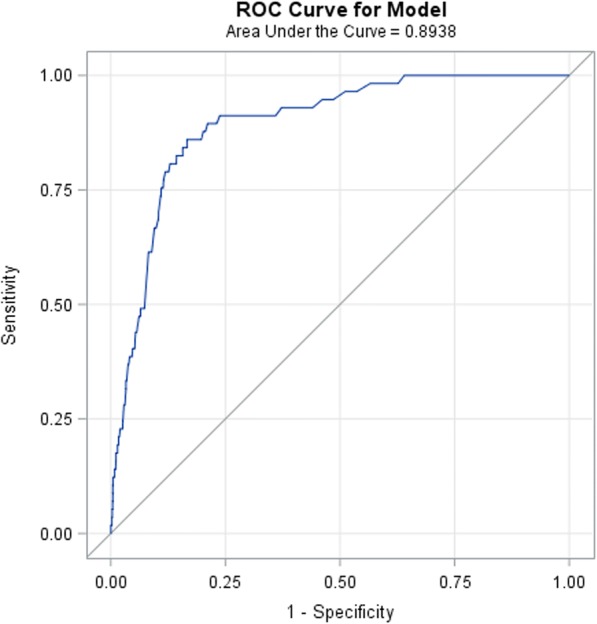
ROC curve of the predictive model on the training data set. AUC (95%CI) = 0.89 (0.86, 0.93)

Univariate logistic regression analysis of the training data showed that cervical dilatation (OR(95%CI): 0.69 (0.51, 0.94)) (for each cm increase), incidence of break through pain (OR(95%CI): 19.74 (10.76, 36.21)), inability to obtain CSF in the CSE technique (OR(95%CI): 6.19 (2.43, 15.78)), post-epidural insertion pain score (OR(95%CI): 1.38 (1.19, 1.59)) (for every one unit increase) and venous puncture (OR(95%CI): 3.85 (1.63, 9.06)) were significantly associated with epidural catheter re-siting. Final multivariable predictive model after stepwise, forward and backward selection method included the following variables—incidence of break through pain (OR(95%CI): 4.42 (3.25, 6.02)), age of parturient (OR(95%CI): 1.07 (1.01, 1.12)) (for each year), artificial rupture of membranes (OR(95%CI): 0.33 (0.1, 1.03)), dinoprostone (Prostin E2) use for induction of labour (OR(95%CI): 0.38 (0.12, 1.18)), spontaneous onset of labour (OR(95%CI): 0.31 (0.10, 0.98)), post-epidural insertion pain score (OR(95%CI): 1.35 (1.14, 1.61)) and problems such as inability to obtain CSF in the CSE technique (OR(95%CI): 2.06 (1.2, 3.53)), venous puncture (OR(95%CI): 1.70 (1.08, 2.68)) and inability to pass catheter through the epidural needle (OR(95%CI): 1.93 (0.65, 5.74)) (Table 2). C-Statistic (95%CI) and AIC for this model was 0.89 (0.86, 0.93) and 545.381 respectively (Fig. 2). Predictive model for epidural catheter re-siting was as follows:

$$ \mathrm{Score}=-5.9334+\left(0.0632\ast \mathrm{Age}\right)+\left(0.3025\ast \mathrm{post}-\mathrm{epidural}\ \mathrm{insertion}\ \mathrm{pain}\ \mathrm{score}\right)+\left(1.4866\ast \mathrm{incidence}\ \mathrm{of}\ \mathrm{break}\ \mathrm{through}\ \mathrm{pain}\right)-\left(1.1134\ast \mathrm{labour}\ \mathrm{onset}\ \mathrm{artificial}\ \mathrm{rupture}\ \mathrm{of}\ \mathrm{membranes}\right)-\left(0.9640\ast \mathrm{labour}\ \mathrm{onset}\ \mathrm{dinoprostone}\ \left(\mathrm{ProstinE}2\right)\right)-\left(1.1744\ast \mathrm{labour}\ \mathrm{onset}\ \mathrm{spontaneous}\right)+\left(0.7232\ast \mathrm{problem}\ \mathrm{of}\ \mathrm{unable}\ \mathrm{to}\ \mathrm{get}\ \mathrm{CSF}\ \mathrm{for}\ \mathrm{CSE}\right)+\left(0.5321\ast \mathrm{problem}\ \mathrm{of}\ \mathrm{venous}\ \mathrm{puncture}\right)+\left(0.6551\ast \mathrm{problem}\ \mathrm{of}\ \mathrm{unable}\ \mathrm{to}\ \mathrm{pass}\ \mathrm{catheter}\right) $$

**Table 2:** Multivariate logistic regression analysis for epidural re-siting predictive risk factors based on internal training data

Risk factors	β coefficients	Standard error (SE)	*P* – value	Adjusted Odds ratio (OR)	95% confidence interval of OR	
					Lower	Upper
Intercept	−5.9334	1.1434	< 0.0001			
Age	0.0632	0.0271	0.0197	1.065	1.0102	1.1233
Post-epidural Pain Score	0.3025	0.0881	0.0006	1.353	1.1388	1.6082
Breakthrough Pain (Ref = No)	1.4866	0.1573	< 0.0001	4.422	3.2485	6.0189
Artificial rupture of membranes (Ref = No)	−1.1134	0.5831	0.0562	0.328	0.1047	1.0298
Dinoprostone insertion for induction of labour (Ref = No)	−0.9640	0.5775	0.0951	0.381	0.123	1.1828
Spontaneous onset of Labour (Ref = No)	−1.1744	0.5877	0.0457	0.309	0.0977	0.9777
Inability to obtain CSF in the CSE technique (Ref = No)	0.7232	0.2746	0.0085	2.061	1.2032	3.5307
Venous puncture (Ref = No)	0.5321	0.2322	0.0219	1.702	1.0802	2.6834
Inability to pass catheter through epidural needle (Ref = No)	0.6551	0.5576	0.2400	1.925	0.6455	5.7431

and $$ \mathrm{estimated}\ \mathrm{probability}=\frac{{\mathrm{e}}^{\mathrm{Score}}}{1+{\mathrm{e}}^{\mathrm{Score}}} $$.

Incidence of breakthrough pain and the parturients’s age had significant roles in the predictive model. C-Statistic (95%CI) and AIC droped to 0.66 (0.58, 0.73) and 650.76, respectively after removing incidence of breakthrough pain from the final multivariate model. Similarly, the C-Statistic (95%CI) and AIC were reduced to 0.86 (0.82, 0.92) and 548.79 respectively after removing parturients’s age. As an example, for a 25 year-old mother who had CSF obtained from the spinal needle during the CSE insertion, experienced a venous puncture during the procedure, whose anaesthesiologist was unable to pass the catheter through the epidural needle, a post-epidural insertion pain score of 5 and who subsequently developed breakthrough pain—she would have score of 87.21 and would have a 70.52% probability of “*requiring epidural catheter re-siting*”.

### Performance in internal and external validation data

**Fig. 3:**
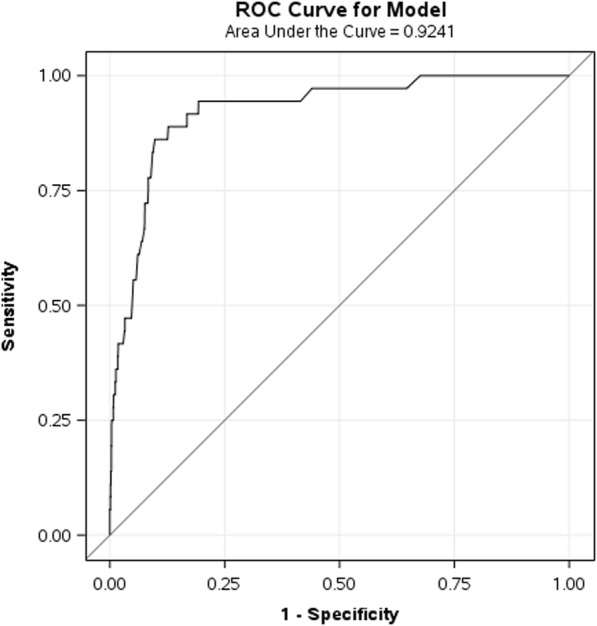
ROC curve of the predictive model on the internal validation data set. AUC (95%CI) = 0.92 (0.88, 0.97)

**Fig. 4:**
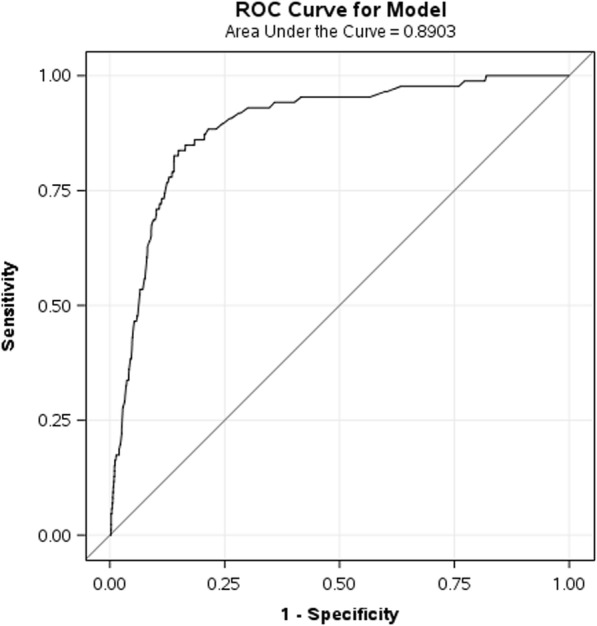
ROC curve of the predictive model on the external validation data set. AUC (95%CI) = 0.89 (0.86, 0.92)

After fitting the above-mentioned predictive model, the internal validation data showed C-statistic (95%CI) as 0.92 (0.88, 0.97) (Fig. 3). Using a threshold for the probability of epidural catheter re-siting of 0.02, the sensitivity and specificity to predict epidural catheter re-siting ranged from 0.73 to 0.96 and 0.85 to 0.87 respectively. Similarly, the C-statistic (95%CI) for external validation data was 0.89 (0.86, 0.92) (Fig. 4). These estimates are very much consistent with the corresponding estimate based on the training data. Performance of model based on C – statistic using 2014–2015 and 2012–2013 data were also provided in Table 3.

**Table 3:** Performance of predictive models based on training, internal and external validation data

Measures	2014–2015		2012–2013
	Training data	Internal validation	External validation
C – statistic	0.894 (0.858, 0.930)	0.924 (0.882, 0.966)	0.890 (0.858, 0.923)

## Discussion

The incidence of epidural catheter re-siting in 2014–2015 in our centre was 0.88%, which was similar to the incidence of 0.85% in 2012–2013 [4], and was lower than the reported incidence in other centres of about 1.6–6.8% [11–13]. The similarity in the incidence of epidural catheter re-siting between the two time periods in our centre could be attributed to our standardized guidelines for managing inadequate epidural analgesia in labour.

### Predictive factors for epidural catheter re-siting

Increasing age, the presence of breakthrough pain, higher pain scores immediately after post-epidural insertion, the inability to obtain CSF in the CSE technique and a venous puncture during the neuraxial blockade were found to be predictive of epidural re-siting, while the spontaneous onset of labour was found to be protective.

The presence of breakthrough pain was the most significant independent risk factor for epidural catheter re-siting (OR(95%CI): 4.42 (3.25, 6.02)). The incidence of breakthrough pain has been reported to range from 12 to 33% [5, 11, 14]. However, the incidence of breakthrough pain was 14.1% in this study, which was similar to the incidence of 14.3% from our previous results [4]. Breakthrough pain has been shown to be associated with multiple factors such as maternal characteristics, obstetric or anaesthetic factors, including an increased body mass index, dysfunctional labour [15] and catheter related issues [16] associated with inadequate analgesia.

The majority of breakthrough pain is managed conservatively with an additional bolus of local anaesthetic with or without fentanyl via the epidural catheter [17]. However, if labour is prolonged, it may be appropriate to re-site the epidural catheter to minimize maternal distress and to reestablish adequate pain relief. Moreover, should a Caesarean section be subsequently required, the sensory blockade can be extended to achieve reliable anaesthesia for surgery. Recurrent breakthrough pain during labour epidural analgesia has been shown to be a predictive factor for failed augmentation for anaesthesia for Caesarean section [18].

In our centre, 93.0% of the labour epidural analgesia administered was using the CSE technique. The onset of pain relief has been shown to be more rapid and more reliable using the CSE technique than plain epidural analgesia alone, and is also less likely associated with recurrent breakthrough pain [8, 19]. A higher pain score immediately after post-neuraxial blockade (OR(95%CI): 1.35 (1.14, 1.61)) could possibly indicate a failure of the CSE technique and the parturient may subsequently then require epidural catheter re-siting. Groden et al. found that the CSE technique is less likely to fail during labour and that the time to detection of a failed catheter was significantly longer compared to epidural only technique, which also gives evidence that CSE is a more reliable technique [20].

Increasing age is associated with increased difficulty of neuraxial blockade [21], which could be due to the calcification of ligaments of the spine and osteophyte formation. This could potentially result in an increased failure rate and increased need for epidural catheter re-siting. Although most parturients are expected to be young, maternal age is increasing. In our cohort, the wide age range of parturients (14 to 48 years) may account for increasing age being an independent predictive factor of epidural catheter re-siting. However, age was not found to be a factor influencing epidural re-siting in another observational study of epidural re-siting in labour analgesia [22].

Labour onset can be spontaneous or facilitated by the insertion of dinoprostone (Prostin E2) or the artificial rupture of membranes [23]. The spontaneous onset of labour (OR(95%CI): 0.31 (0.1, 0.98)) was identified to be an independent protective factor for epidural catheter re-siting. However, these are not mutually exclusive—a parturient may have spontaneous onset of labour and yet also had her membranes ruptured artificially. Nonetheless, these factors were included in the multivariate predictive model for epidural catheter re-siting.

Technical problems during the procedure have also been identified to be independent risk factors for epidural catheter re-siting. During a CSE insertion, after the epidural space has been identified with a loss of resistance, insertion of the spinal needle through the back-eye of the epidural needle may not attain a dural puncture to administer the spinal component. Because the dural sac is triangular-shaped at the lumbar level with the base directed anteriorly, if the epidural needle is not placed in the midline, the spinal needle would not puncture the dura if placed off midline [24]. A plain epidural technique may be performed instead and the CSE procedure abandoned to minimize distress from another neuraxial attempt. However, an epidural catheter that is inserted in conjunction with a CSE technique has a greater likelihood of being in the correct space and providing adequate pain relief [25]. Our findings are consistent with another study that found that CSEs were less likely to be re-sited compared to a plain epidural technique for labour analgesia [22]. Thus, the inability to obtain CSF during the CSE technique is a predictive risk factor of epidural catheter re-siting—this could be associated with inadequate pain relief. At our centre, the landmark technique is the predominant method used during epidural catheter insertion; there is recent evidence that ultrasound guidance could reduce the rate of epidural resiting and decrease the number of attempts by junior residents [26].

Venous puncture during neuraxial blockade placement is predictive for epidural catheter re-siting. Intravenous placement of the epidural catheter can occur in 5–7% [11] of labour epidurals and is more common in parturients because of a distended venous plexus from compression of the gravid uterus [27]. Venous placement of the epidural catheter may later present as a non-working labour epidural or systemic toxicity [27]. The methods that could reduce the risk of venous puncture include positioning the patient in a lateral position as opposed to sitting during epidural catheter placement, administration of fluid through the epidural needle before threading the epidural catheter into the space and using single-orifice catheters [28].

### Strengths of study

Epidural catheter re-siting in parturients can be distressing and has also been found to be associated with maternal dissatisfaction [29]. The robustness of our predictive model (C-statistic of 0.89, 0.92 and 0.89 when used on the training, internal validation and respectively) enables the anaesthesiologist to reliably identify and target modifiable risk factors that predict epidural catheter re-siting. This could potentially decrease the likelihood of epidural catheter re-siting, thereby improving maternal satisfaction. Moreover, the early identification of such parturients so that a higher epidural catheter re-siting risk can be communicated among the clinicians in a timely manner.

Another strength of the study is the large number of patients that were included in the cohort, which is advantageous for the uncommon outcome of epidural catheter re-siting. We also externally validated the multivariate model with the predictive factors for epidural catheter re-siting with our previous 2012–2013 data, which increased the robustness of the model and the validity of the results. To our knowledge, there have been no other large scale studies that have developed such a predictive model.

### Limitations of study

The limitations of this retrospective cohort study could be related to selection bias and confounding factors. A prospective cohort study, although ideal, will be logistically challenging because of the high patient workload of about 5000 labour epidural analgesia every year at our centre [4]. Although a standardised neuraxial labour analgesia form was used, there was still a small number of missing data in some variables [4]. These were excluded from analysis and the data was cross-checked if the values were extreme outliers. Labour epidural analgesia was also performed by multiple anaesthesiologists and charting was also performed by different midwives, who may differ in their reporting. However, guidelines on managing labour epidural analgesia at our centre remained unchanged in the two periods which was a continuum. The anesthesiologists were different in the two time periods because anaesthesiology residents rotated through our centre for training. Nonetheless, this was not found to be a predictive risk factor for epidural catheter re-siting.

Our previous study identified independent risk factors that were associated with epidural catheter re-siting. The associated factors included both variables obtained prior and post to the epidural catheter re-siting procedure. In this study, we only chose those variables available prior to the epidural catheter re-siting procedure with *p*–value < 0.3 on the univariate analysis or that were clinically meaningful to develop the predictive model for epidural catheter re-siting. The exclusion of factors that occurred after epidural catheter re-siting (e.g. need for Caesarean section for delivery etc.) may have possibly changed the weightage of the other factors in this predictive model, thus accounting for the differences seen in the factors identified between the current and previous study.

## Conclusion

In summary, we have developed and validated a predictive model for epidural catheter re-siting for labour analgesia. The knowledge of such factors helps identify parturients at high risk of epidural catheter re-siting early, thereby allowing timely targeted management. The predictive model could be further externally validated and refined with recent data from our electronic labour neuraxial analgesia database to improve its robustness.

## Abbreviations

AIC: Akaike information criteria
AUC: Area under curve
CSE: Combined spinal-epidural
CSF: Cerebrospinal fluid
OR: Odds ratio
ROC: Receiver operator characteristic
SD: Standard deviation
